# Associations Between Microaggressions, Discrimination, and Brain Health Among Black Women Living with HIV

**DOI:** 10.1007/s40615-026-03003-0

**Published:** 2026-05-11

**Authors:** Michael Robinson, Valerie Daniel, Reyanna St Juste, Ashley Yankulin, Layomi Adeojo, Lalitha Kanumuri, Mya Wright, Maria Fernanda Silva, Rachelle Reid, Naysha Shahid, Victoria Petrulla, Simon Howard, Sannisha K. Dale

**Affiliations:** 1 Department of Psychology, University of Miami, 5665 Ponce de Leon Blvd, Miami, FL 33146, USA

**Keywords:** Black women, HIV, Brain health, Health equity

## Abstract

**Introduction:**

Despite advancements in the treatment of HIV, Black women living with HIV (BWLWH) experience significant disparities in brain health and related factors, which may be linked to microaggressions, systemic inequities, and discrimination. Factors such as functional activity, brain health, sleep, and diet also play a role in the overall health of an individual. This study examines how experiences of microaggressions are associated with brain health-related factors among BWLWH.

**Methods:**

Utilizing cross-sectional data from 183 BWLWH, this study employed linear regressions to assess relationships between psychosocial stressors (microaggressions, discrimination) and brain health-related measures including the Functional Activities Questionnaire, The Healthy Brain 9, and Sleep Evaluation (night-time, treatments, and behaviors).

**Results:**

Findings indicate that a higher frequency of HIV-related microaggressions (total direct and indirect) and gendered racial microaggressions were associated with cognitive function difficulties and sleep disturbances. Women who reported higher experiences of race-, gender-, and HIV-based discrimination also had cognitive function difficulties and sleep disturbances, but only race- and gender-related discrimination were related to lower global health ratings.

**Conclusion:**

This study highlights the role microaggressions and discrimination may play in brain health and related factors among BWLWH. Addressing these disparities requires targeted interventions that promote health equity, increase access to culturally competent care, and integrate community-based resilience strategies to support cognitive well-being.

## Introduction

Black women living with HIV (BWLWH) face significant disparities in brain health outcomes due to the compounded effects of structural inequities, systemic discrimination, and economic instability [1]. National surveillance data indicate that Black women account for a disproportionate number of new HIV diagnoses and HIV-related mortality in the United States. In 2021, Black women constituted 50% of all US women diagnosed with HIV, despite representing only 13% of the female population [2]. Similarly, in 2021, African American women comprised 60% of HIV diagnoses in Florida, highlighting pronounced regional disparities [3].

The intersection of racism, sexism, and HIV stigma places Black women at elevated risk for adverse health outcomes, including cognitive decline and mental health distress [1]. Chronic stress resulting from discrimination and marginalization contributes to elevated allostatic load—a physiological response to prolonged stress exposure—linked to cognitive impairment and other negative health outcomes [4, 5]. Furthermore, exposure to adverse childhood experiences (ACEs) and trauma in adulthood further complicates the health trajectory of BWLWH [1]. Chronic stress has been shown to accelerate biological aging and heighten vulnerability to neurocognitive decline [6]. Moreover, the stress of navigating multiple marginalized identities has been associated with higher rates of depression and anxiety, which are independently linked to poorer cognitive functioning over time [7]. Additionally, systemic barriers to healthcare, such as provider bias and medical mistrust, limit early detection and timely intervention for cognitive decline [8]. Medical mistrust, rooted in historical and ongoing systemic racism, has been identified as a critical factor influencing participation in HIV health services among Black women in the U.S. South [9].

Intersectionality theory offers a valuable framework for understanding how multiple forms of oppression—such as racism, sexism, and HIV stigma—converge to shape the lived experiences of BWLWH. Rooted in a long tradition of Black feminist thought, intersectionality draws from activism and scholarship including the Combahee River Collective [10], Audre Lorde, Patricia Hill Collins, and Kimberlé Crenshaw, who formally coined the term to describe the interconnected nature of social categorizations and systemic oppression [11]. Addressing these disparities requires comprehensive strategies that tackle the root causes of structural inequities and promote equitable access to healthcare, education, and economic opportunities [12].

Racism operates as a chronic and pervasive stressor that contributes to brain health disparities by increasing allostatic load and impairing neurocognitive functioning over time [13, 14]. Racial discrimination has been consistently associated with poorer sleep quality, diminished global health ratings, and reduced cognitive performance among Black populations [15]. Repeated exposure to racial bias activates physiological stress pathways—such as the hypothalamic-pituitary-adrenal (HPA) axis— can negatively affect memory, attention, and executive functioning [13]. These effects are compounded by socioeconomic disadvantage and systemic inequities in healthcare access, creating an even greater burden on brain health [16]. For BWLWH, these consequences are amplified as racism intersects with both gender and HIV status, introducing additional barriers to care and well-being [17].

Sexism and gender-based discrimination are also important predictors of negative brain health outcomes, particularly for women who experience multiple, intersecting forms of marginalization [18]. Gender discrimination has been associated with disrupted sleep, impaired cognitive functioning, and poorer overall health outcomes [19, 20]. Among BWLWH, gender-based mistreatment can manifest in various ways—including being overlooked in clinical settings, facing harmful assumptions about sexuality, or being held to unrealistic expectations of strength and resilience [21]. These experiences contribute to increased cognitive load and emotional exhaustion [22], raising the risk for sleep disturbances and impaired functional health [23]. Importantly, these stressors are often normalized or dismissed, allowing their psychological and physiological consequences to go unaddressed [24].

HIV-related stigma and discrimination remain persistent challenges for individuals living with HIV and are strongly linked to adverse brain health outcomes [25]. Research has demonstrated that HIV-related discrimination is associated with lower cognitive functioning, more frequent sleep disruptions, and poorer global health assessments [26, 27]. Enacted HIV stigma—manifesting through direct discrimination, bias, and mistreatment from healthcare providers, community members, and broader institutions—poses significant barriers to the health and well-being of Black women living with HIV [28]. In response to these repeated experiences of enacted stigma, many women may also begin to internalize negative beliefs about themselves, anticipate mistreatment in healthcare settings, and experience heightened social isolation [29]. For Black women, HIV stigma intersects with race and gender oppression, creating a uniquely stressful environment that intensifies neurocognitive risk [30]. Moreover, fear of judgment or discrimination can prevent individuals from seeking timely and supportive care, further exacerbating health disparities [31].

Microaggressions and discrimination operate across multiple levels, including interpersonal (micro-level) and structural (macro-level) domains [32]. Microaggressions reflect subtle, everyday interpersonal expressions of bias, whereas discrimination captures broader systemic and institutional inequities that shape access to resources, opportunities, and care [33]. Understanding how these multilevel stressors interact is critical for identifying pathways through which social adversity contributes to disparities in brain health among Black women living with HIV.

In addition to structural oppression, individual health behaviors such as physical activity, diet, and cognitive engagement play an essential role in supporting brain health [34]. Studies have shown that regular participation in cognitively stimulating activities, maintaining a nutritious diet, and engaging in consistent physical activity are protective against cognitive decline [35]. However, BWLWH often face barriers to accessing resources that support these health-promoting behaviors due to systemic inequities [36]. Structural oppression directly impacts opportunities for physical activity and healthy eating, as many Black women live in neighborhoods with limited access to affordable nutritious foods (food deserts) and environments that discourage physical activity—such as the absence of sidewalks, lack of green spaces, and concerns about neighborhood safety [37]. Economic instability, caregiving responsibilities, and limited healthcare access create obstacles to prioritizing preventive care [37]. In addition, chronic illness—often compounded by the lack of culturally competent healthcare—further limits opportunities to focus on brain health [38]. While psychosocial resilience, strong social networks, and self-efficacy have been identified as protective factors against chronic stress, these strengths remain underexplored in the context of brain health disparities among BWLWH [35–37].

This study aimed to expand the literature and address gaps by examining the relationship between intersectional adversity and brain health outcomes among Black women living with HIV. By assessing the interplay of discrimination, this research sought to provide a more comprehensive understanding of the factors influencing cognitive function, mental well-being, and overall health in this population. Findings from this study can inform culturally responsive interventions that incorporate social, economic, and psychological support systems to promote brain health equity in this population. A comprehensive understanding of the intersections of racism, sexism, classism, and HIV stigma in shaping brain health outcomes will be critical in developing policies and interventions that foster resilience and improve health equity among BWLWH.

## Materials and Methods

### Participants and Procedures

From November 2023 through March 2024, 183 BWLWH enrolled in a longitudinal cohort study completed a standard assessment battery (given every 3 months) as well as a one-time interviewer-administered psychosocial assessment focused on brain health-related factors. Inclusion criteria for the parent study included: (a) being a cisgender Black woman, (b) able to give consent, (c) 18 years of age or older, (d) able to speak and understand English (e) living with HIV, and (f) owns a cell phone to receive one daily text message. Trained study staff allowed time for participants to read the consent form and then discussed the form with each participant. For their time and effort women were given $75 for the standard assessment and $40 for the brain health assessment. All study procedures received approval from the University of Miami Institutional Review Board prior to study onset.

### Measures

#### Sociodemographic Characteristics

This survey gathered a range of personal and demographic details from participants, such as their age, educational background, place of birth, yearly income, employment status, sexual orientation, relationship situation, number of children, living arrangements, religious affiliation, and HIV viral load status—specifically noting whether their viral load was detectable or undetectable. Additionally, the survey included questions about their current health insurance coverage.

#### Adversity Indicators

In this study, adversity indicators were conceptualized as reflecting related but distinct forms of social harm. Microaggressions were defined as subtle, everyday interpersonal slights, indignities, or exclusions, whereas discrimination was defined as broader enacted unfair treatment across institutional and interpersonal contexts. This distinction guided the selection of measures assessing everyday slights, recurring interpersonal bias, and more overt forms of identity-based mistreatment.

##### Multiple Discrimination Scale

To evaluate experiences of discrimination across multiple identity categories, this study employed the Multiple Discrimination Scale [39], consisting of 52 items in total—39 from the original scale and 13 additional questions adapted by the authors to specifically address gender-based discrimination. The scale assessed enacted discrimination related to race, HIV status, gender, and sexual orientation, using 10 parallel items for each category. Participants were asked to report whether they had encountered specific forms of unfair treatment, such as institutional exclusion, within the past 12 months. There was 0% missing data for the sexual orientation and gender categories and 0.5% missing data for the HIV status and race categories. Responses were recorded as either “yes” (coded as 1) or “no” (coded as 0). Responses were recorded as either “yes” (coded as 1) or “no” (coded as 0). Example items include questions like, “Have you been denied or lost housing because of your race?” or “Have you been refused employment or terminated from a job due to your HIV status?” This approach allowed for a detailed exploration of how various forms of discrimination manifest across different aspects of identity. This measure has been used in prior research with Black populations and individuals living with HIV and was selected because of its relevance for assessing enacted discrimination across multiple identity domains [22].

##### Gendered Racial Microaggressions

This 26-item questionnaire is designed to capture the distinct microaggressions experienced by Black women as a result of the intersection of racial and gender bias [39]. It assesses both how often these encounters occur and the degree of stress they cause (i.e. appraisal). The scale is organized into four thematic dimensions: assumptions related to appearance and sexualization, experiences of being ignored or excluded, expectations to be perpetually strong, and being unfairly labeled as angry. Example items include experiences such as being called angry while speaking calmly, being told they are overly independent, or receiving negative remarks about their skin tone. Participants indicate how frequently they have encountered each situation in their lifetime, ranging from 0 (never) to 5 (once a week or more), and separately rate how emotionally distressing each experience was (e.g., 0 [this has never happened to me], 1 [not stressful at all]. 5 [extremely stressful]). In terms of missing data, the overall frequency score was missing 1.1% of data. Appraisal was not assessed for women (33.9%) who indicated that they did not experience a type of microaggression. This tool provides valuable insight into the cumulative impact of racial and gendered microaggressions on Black women. This measure has been widely used in studies of Black women and was selected because it captures the intersection of racialized and gendered interpersonal bias that is especially relevant to the lived experiences of Black women [40].

#### Measures for Brain Health-related Factors

In this study, brain health was operationalized as a multidimensional construct encompassing cognitive functioning, daily functional ability, sleep quality and sleep-related disturbances, and self-perceived global health. The measures below were therefore selected to capture multiple dimensions of brain health relevant to populations affected by HIV, including cognitive screening performance, functional ability, sleep-related symptoms, and perceived global well-being. Together, they provide a broader assessment of neurocognitive and behavioral functioning than any single measure alone.

##### Global Health Ratings

The Global Health Ratings is a brief self-evaluation instrument that asks individuals to reflect on four key dimensions of their well-being: physical health, mental health, emotional state, and social connectedness. For each domain, participants assign a rating using a 4-point scale, with 1 representing “Poor” and 4 representing “Excellent.” This measure offers a snapshot of how participants perceive their current overall health, allowing for a concise yet holistic understanding of their general wellness across multiple aspects of life. There was 0% missing Global Health Rating data.

##### Functional Activities Questionnaire (Social Engagement Scale)

The Functional Activities Questionnaire (Social Engagement Scale) evaluates a person’s ability to carry out everyday tasks over the preceding month. It measures functional independence in areas such as handling money matters (like paying bills or balancing a checkbook), keeping documents in order, shopping for basic necessities on their own, participating in leisure activities, cooking meals, and navigating transportation beyond their immediate surroundings. Each task is rated on a four-level scale: 1 for activities performed normally or never undertaken, 2 for tasks completed independently but with some difficulty, 3 for tasks requiring help, and 4 for complete dependence on others. This tool offers a clear overview of a person’s functional capabilities and highlights areas where support or intervention may be needed to maintain independence. There was 0% missing on the Functional Activities Questionnaire.

##### The Healthy Brain 9 (HB9)

The Healthy Brain 9 (HB9) is a self-report measure designed to capture perceived shifts in cognitive and functional abilities over a five-year period. Participants assess their current capacity in comparison to how they functioned in the past, using a scale from 0 (“No Change: I’m still able to do this as before”) to 4 (“Significant Change: I now struggle to do this and require help”). The questionnaire explores a broad range of cognitive and everyday tasks, such as recalling recent information, managing appointments and medications, making decisions, handling finances, remembering dates, and navigating routine responsibilities like work, errands, and social participation. It also examines communication skills, household management, independent travel, and engagement in hobbies. By evaluating changes across these domains, the HB9 offers a snapshot of cognitive aging or decline and can help identify areas where individuals may benefit from support or intervention. There was 0% missing Healthy Brain 9 data.

##### Sleep Evaluation

The Sleep Evaluation tool captures information about participants’ sleep habits at night, their perceived quality of rest, and experiences with daytime drowsiness over the past month. Participants respond based on their own observations as well as any input from others, such as partners or household members. Nighttime questions explore challenges like difficulty falling asleep, waking frequently, rising earlier than intended, morning fatigue, and feeling sleep deprived. Each response is rated on a scale from 0 (“Never”) to 3 (“Often”). Participants also rate their overall sleep quality using a scale ranging from “Excellent” to “Poor.” To assess daytime sleepiness, the questionnaire includes items about dozing off in passive situations—like watching television, sitting quietly, or riding in a car. These are rated on the same 0 to 3 scale. Together, these questions offer a broad picture of sleep disturbances and daytime fatigue. There was 0% missing Sleep Evaluation data.

##### Sleep Behaviors

The Sleep Behaviors questionnaire examines a range of actions and physical experiences that may influence sleep quality. Participants indicate either “Yes” or “No” to a series of items that inquire about behaviors such as loud breathing or snoring, sudden gasping or breathing interruptions during sleep, frequent nightmares or intense dreaming, and physical activity during sleep like talking, kicking, or punching. It also explores sensations of restlessness or tingling in the legs, involuntary leg movements, and teeth grinding. This instrument helps identify patterns that could disrupt restful sleep or signal underlying sleep disorders. There was 0% missing Sleep Behaviors data.

##### Sleep Treatments

The Sleep Treatments questionnaire collects data on participants’ experiences with various methods used to address sleep-related concerns. Individuals respond with “Yes” or “No” to items asking whether they have undergone an overnight sleep evaluation, received a diagnosis for a sleep disorder (and what type, if applicable), taken non-prescription sleep aids, or used nighttime devices such as sleep machines. This tool is designed to capture the range of interventions participants have utilized to manage or improve their sleep health. There was 0% missing Sleep Treatments data.

##### Montreal Cognitive Assessment (MoCA)

The Montreal Cognitive Assessment (MoCA) is a brief screening tool designed to detect mild cognitive impairment across a range of domains. It assesses functions such as attention and concentration, executive functioning, memory, language, visuospatial skills, abstract thinking, and orientation. The assessment consists of various tasks, including word recall, clock drawing, naming objects, and digit span tests, providing a comprehensive snapshot of cognitive abilities. Scores range from 0 to 30, with a score of 26 or above generally considered normal. There was 3.8% missing MoCA data in this dataset. The MoCA helps identify early cognitive decline that might otherwise go unnoticed and serves as a valuable guide for determining whether further evaluation or intervention is needed.

#### Covariates

Existing research associating mental health and HIV medication adherence with intersectional adversities (e.g., microaggressions and discrimination) [41] and brain health factors [42] suggests that these are key covariates to include.

## Mental Health

Participant mental health and substance use was measured using a semi-structured clinical interview administered by master’s level clinicians under the supervision of the study principal investigator (a licensed clinical psychologist). For the purpose of this study, we utilized participant current diagnosis at the time of their study visit where they completed all other measures. Participants who indicated for one or more mental health diagnoses were assigned a value of 1 for having a mental health diagnosis, and those who did not have any current mental health diagnoses were assigned a value of 0.

## Medication Adherence

Participants self-reported their HIV medication adherence by responding to the following question: “In the last 30 days, how good a job did you do at taking your medication in the way you were supposed to?” The response scale ranged from “Very poor” (1) to “Excellent” (6). For the purpose of this study, those who reported that they adhered to their HIV medications as “Good”, “Very Good”, or “Excellent” were assigned a value of 1. The participants who reported “Fair,” “Poor,” or “Very Poor” levels of HIV medication adherence were assigned a value of 0.

### Data Analysis

Linear regression analyses were conducted in R Studio to examine associations between psychosocial stressors (microaggressions and discrimination) and brain health-related outcomes while controlling for mental health diagnosis and HIV medication adherence. Separate models were run for each intersectional adversity and brain health-related variable. Due to the cross-sectional nature of our analyses, findings were indicative of associations rather than causation. Statistical significance was evaluated at *p* <.05. The following sections describe the findings in detail, organized by thematic clusters.

## Results

As displayed in (Table 1), the age range of participants was 26 to 85 years, with an average age of 56.68 and a median age of 59. Educational attainment among participants showed that approximately 31.69% of women completed high school/GED. Employment and educational engagement status revealed that 57.37% had an annual household income of less than $11,999, 14.22% were engaged in full or part-time work, and 63.39% were on disability.

### Microaggressions and Brain Health Factors

#### Gendered Racial Microaggression and Brain Health Factors

Regression analyses were conducted to examine the relationship between gendered racial microaggressions and various health and functional outcomes. Frequency of experiencing gendered racial microaggressions, covaried with mental health diagnosis status and medication adherence, was significantly associated with Healthy Brain-9 scores, *F*(3, 161) = 3.95, *p* <.01 (Table 2), where higher frequency of gendered racial microaggressions was associated with decreased cognitive and daily functioning (b = 1.53, *p* <.05). Similarly, frequency of gendered racial microaggressions was significantly associated with night sleep scores, *F*(3, 161) = 4.53, *p* <.05 (Table 2), indicating that participants who reported more frequent gendered racial microaggressions also experienced poorer sleep during the night (b = 0.89, *p* <.05). Frequency was also significantly associated with sleep treatment scores, *F*(3, 161) = 6.05, *p* <.05 (Table 2), with higher reported frequency corresponding to increased usage of sleep aids or treatments (b = 1.75, *p* <.05).

Appraisal of gendered racial microaggressions was also examined in relation to multiple domains. Gendered racial microaggression appraisal was significantly associated with Healthy Brain-9 scores, *F*(3, 107) = 3.82, *p* <.05 (Table 3), such that higher appraisal was associated with diminished everyday functioning (b = 1.67, *p* <.01) (Table 3). Night sleep scores were also significantly associated with appraisal, *F*(3, 107) = 5.14, *p* <.05 (Table 3), indicating that greater microaggressions related distress was linked to poorer sleep quality (b = 0.69, *p* <.05). Additionally, appraisal was significantly associated with sleep treatment scores, *F*(3, 107) = 9.54, *p* <.001 (Table 3), with higher appraisal associated with increased reliance on sleep treatments (b = 2.39, *p* <.001) (Table 3).

### HIV-Related Microaggressions and Brain Health Factors

Regression analyses were conducted to explore the associations between HIV-related microaggressions—total, direct, and indirect—and a range of health and behavioral outcomes. Total HIV-related microaggressions were significantly associated with Healthy Brain-9 scores, *F*(3, 162) = 6.93, *p* <.001 (Table 4), such that higher reported experiences were associated with reduced cognitive and functional performance (b = 1.96, *p* <.05). Total HIV-related microaggressions also significantly related to night sleep scores, *F*(3, 162) = 7.89, *p* <.001 (Table 4), indicating poorer sleep with greater exposure (b = 1.22, *p* <.001) (Table 4). Additionally, sleep treatment scores were significantly associated, *F*(3, 162) = 7.43, *p* <.01 (Table 4), suggesting increased reliance on sleep aids (b = 1.88, *p* <.01).**) (Table 4).

Direct HIV-related microaggressions were also significantly associated with multiple outcomes. Specifically, direct HIV microaggressions significantly related to night sleep scores, *F*(3, 162) = 7.29, *p* <.001 (Table 5), indicating greater nighttime sleep disruption (b = 1.13, *p* <.001) (Table 5). Sleep treatment scores were also significantly associated, *F*(3, 162) = 7.09, *p* <.01 (Table 5), with higher exposure associated with increased treatment usage (b = 1.74, *p* <.01) (Table 5).

Indirect HIV-related microaggressions were similarly associated with multiple outcomes. Indirect microaggressions significantly related to Healthy Brain-9 scores, *F*(3, 162) = 4.19, *p* <.05 (Table 6), such that higher levels were associated with reduced cognitive functioning (b = 1.23, *p* <.05). Night sleep scores were also significantly associated, *F*(3, 162) = 6.40, *p* <.01 (Table 6), indicating poorer sleep quality (b = 0.95, *p* <.01) (Table 6). Additionally, sleep treatment scores were significantly related, *F*(3, 162) = 6.29, *p* <.05 (Table 6), suggesting increased reliance on sleep aids (b = 1.38, *p* <.05).

### Discrimination and Brain Health Factors

#### Racism and Brain Health Factors

Regression analyses were conducted to assess the relationship between racial discrimination, as measured by the racial discrimination subscale of the Multi-Discrimination Scale (MDS), and several health and behavioral outcomes. The MDS racism subscale significantly related to global health scores, *F*(3, 161) = 9.16, *p* <.01 (Table 7), such that higher levels of racial discrimination were associated with lower overall global health (b =−0.10, *p* <.01) (Table 7). The MDS racism subscale also significantly related to Functional Activities Questionnaire scores, *F*(3, 161) = 4.32, *p* <.05 (Table 7), indicating decreased daily functioning with greater discrimination (b = 0.71, *p* <.05).

Sleep-related outcomes were similarly associated with racial discrimination. Night sleep scores were significantly related, *F*(3, 161) = 4.09, *p* <.05 (Table 7), indicating poorer sleep quality (b = 0.45, *p* <.05). Sleep behavior scores were also significantly associated, *F*(3, 161) = 6.12, *p* <.01 (Table 7), suggesting more irregular sleep patterns (b = 0.30, *p* <.01) (Table 7). Finally, sleep treatment scores were significantly related, *F*(3, 161) = 5.63, *p* <.05 (Table 7), with higher discrimination associated with greater reliance on sleep treatments (b = 0.88, *p* <.05). (Table 7).

### Gender-Related Discrimination and Brain Health Factors

Regression analyses were conducted to examine the relationship between gender-based discrimination and health outcomes. The gender-based discrimination subscale significantly related to global health scores, *F*(3, 162) = 8.35, *p* <.05 (Table 8), such that higher discrimination was associated with lower overall global health (b =−0.10, *p* <.05). Functional Activities Questionnaire scores were also significantly related, *F*(3, 162) = 5.22, *p* <.01 (Table 8), indicating decreased daily functioning (b = 1.03, *p* <.01) (Table 9).

Sleep-related outcomes were also significantly associated with gender discrimination. Night sleep scores were significantly associated, *F*(3, 162) = 4.82, *p* <.05 (Table 8), suggesting poorer sleep (b = 0.65, *p* <.05). Sleep behavior scores were significantly related, *F*(3, 162) = 5.12, *p* <.05 (Table 8), indicating more disrupted sleep patterns (b = 0.30, *p* <.05). Additionally, sleep treatment scores were significantly related, *F*(3, 162) = 5.92, *p* <.05 (Table 8), reflecting increased reliance on sleep aids (b = 1.12, *p* <.05). **).

### HIV-Related Discrimination and Brain Health Factors

Regression analyses were conducted to assess the impact of HIV-related discrimination, as measured by the HIV subscale of the Multi-Discrimination Scale (MDS), on health and cognitive outcomes. HIV-related discrimination was examined in relation to global health, daily functioning, and cognitive performance. The MDS HIV subscale was not significantly related to global health scores (b = −0.04, *p* = .150 (Table 9). However, HIV-related discrimination significantly related to Functional Activities Questionnaire (FAQ) scores, *F*(3, 161) = 5.56, *p* <.01 (Table 9), such that greater experiences of HIV-related discrimination were associated with poorer daily functioning (b = 0.75, *p* <.01) (Table 9). In addition, the MDS HIV subscale significantly related to Montreal Cognitive Assessment (MoCA) scores, *F*(3, 161) = 3.03, *p* <.05 (Table 9), indicating that higher levels of HIV-related discrimination were associated with lower cognitive performance (b =−0.65, *p* <.05). These findings suggest that HIV-related discrimination was associated with poorer functional and cognitive outcomes, though it was not significantly related to global health in the present analyses.

## Discussion

In this study, we sought to understand the relationship between intersectional adversity and indicators of brain health among BWLWH. Utilizing an intersectional framework, we examined how discrimination and microaggressions targeting race, gender, and HIV status may impact cognitive functioning, sleep quality, and day-to-day functioning. HIV-associated neurocognitive disorders (HAND) remain prevalent even in the era of effective antiretroviral therapy and are characterized by impairments in cognitive, behavioral, and everyday functioning domains [27]. Further, emerging literature suggests that comorbid conditions such as sleep disturbances, chronic pain, and psychosocial stress may contribute to the development and exacerbation of HAND by influencing inflammation, neurotoxicity, and overall cognitive outcomes [27]. However, there is a dearth of literature exploring the associations among neurocognitive factors and intersectional adversities of salience to the lived experiences of BWLWH, who represent the largest portion of women living with HIV in the U.S. Our findings emphasize the potential cumulative toll of these psychosocial stressors, reinforcing the need for multifaceted interventions that address both individual and structural drivers of health inequity.

Discrimination emerged as a consistent and significant factor associated with multiple health outcomes. Racial discrimination was associated with lower global health ratings, greater functional limitations in daily activities, and poorer sleep outcomes. These sleep-related challenges included decreased sleep quality, increased irregularities in sleep behavior, and greater reliance on sleep treatments. The widespread influence of racial discrimination on both physical and cognitive domains supports existing frameworks on minority stress and underscores how chronic exposure to racism shapes neurological and behavioral health trajectories [12]. Racialized stress may operate through mechanisms such as heightened cortisol levels, autonomic nervous system dysregulation, and impaired sleep cycles—each of which has been associated with long-term cognitive decline [13].

Gender-based discrimination followed a similar pattern. Higher reports of gender discrimination were linked to poorer global health, reduced functional capacity, and pronounced disruptions in sleep behavior. Participants experiencing greater gender-related discrimination were more likely to report inconsistent sleep patterns and a need for sleep treatments, pointing to the physiological and emotional burden of navigating sexism, especially in the context of living with a chronic health condition. These findings, consistent with existing literature [43], highlight the importance of understanding how gender-based stress uniquely contributes to cognitive and functional decline. Gender-based discrimination may also be more subtle or normalized in healthcare settings, leading to delays in diagnosis, miscommunication, or internalized stress—all of which can contribute to cognitive strain [44].

HIV-related discrimination was also significantly associated with diminished brain health and daily functioning. Women reporting higher levels of HIV-related discrimination showed increased difficulty with functional activities and lower cognitive performance, as assessed by the Montreal Cognitive Assessment (MoCA). Notably, microaggressions specific to HIV status—both direct (e.g., explicit stigma or mistreatment) and indirect (e.g., subtle slights or exclusion)—were associated with sleep-related challenges. These included decreased sleep quality at night, irregular sleep behavior, and increased use of sleep treatments. Such patterns suggest that living under the constant threat of HIV-related stigma may produce both psychological and biological disruptions, particularly in sleep and cognitive functioning. This stress may be compounded by healthcare avoidance or fear of disclosure, both of which can further limit access to supportive services and contribute to isolation [25].

Gendered racial microaggressions—those rooted in the intersection of racism and sexism—may also have a measurable impact. The frequency of these microaggressions was linked to poorer scores on the Healthy Brain-9, a marker of cognitive functioning and perceived brain health, as well as decreased sleep quality and increased use of sleep interventions. The appraisal of these microaggressions, or the extent to which they were distressing or impactful, was further associated with functional limitations, disrupted sleep, and lower cognitive health scores. These findings illuminate the psychological wear that results not just from the frequency of discriminatory events, but from their emotional toll [45]. This statement is supported by prior literature [46] demonstrating that the appraisal of stressors, such as microaggressions, contributes significantly to cumulative physiological and functional impairments [47] emphasize that the emotional impact of discrimination exacerbates disruptions in mental and physical health [43] link perceived stress to declines in sleep quality and cognitive functioning, while [48] further identify the psychological burden of discriminatory experiences as a key social determinant of cognitive and mental health outcome. Importantly, the impact of appraisal underscores the role of subjective stress in mediating physiological outcomes suggesting that how stressful the harm feels may intensify the biological effects of external stressors [49]. In line with these findings, cognitive performance and daily functioning were closely linked. Women who reported more difficulty with everyday tasks also tended to have lower cognitive scores, indicating that declines in brain health have tangible effects on quality of life and autonomy. These impairments may limit one’s ability to maintain employment, manage medications, or engage in social activities—further compounding health inequities. Moreover, the consistency of these associations across multiple forms of discrimination and microaggressions supports the view that intersectional stigma contributes to a shared pathway of psychosocial stress, physiological burden, and neurocognitive vulnerability [48]. This convergence reflects how compounded marginalization may lead to disproportionate wear and tear on both the body and brain over time [6]. Collectively, these results reinforce the need for intersectional, trauma-informed, and culturally responsive approaches to healthcare and community support [47].

The pervasive impact of discrimination across identity domains and life contexts demands that interventions move beyond symptom management to directly address the structural and social conditions that sustain health inequities. This includes targeting upstream determinants such as healthcare access, provider bias, housing instability, and economic insecurity, all of which shape both stress exposure and the capacity to engage in health-promoting behaviors. Interventions that operate at multiple levels including individual, interpersonal, and structural levels are likely to be most effective in reducing the cumulative burden of psychosocial stress and its downstream neurocognitive consequences [34, 37].

Community based programs that prioritize safety, belonging, and empowerment can play a critical role in improving brain health outcomes for Black women living with HIV [50]. These programs may be particularly effective when they incorporate peer led models, which leverage shared lived experience to foster trust, increase engagement in care, and reduce medical mistrust [9, 50]. In addition, culturally affirming interventions that explicitly acknowledge and address intersectional stigma can help mitigate the internalization of discrimination and reduce chronic stress burden [29, 30]. Models that integrate trauma informed care by emphasizing safety, trustworthiness, choice, collaboration, and empowerment can further buffer the physiological and psychological effects of repeated stress exposure [35, 44, 47].

Importantly, these approaches may influence brain health through several interrelated pathways, including reductions in stress related physiological activation such as dysregulation of the hypothalamic pituitary adrenal axis, improvements in sleep quality, enhanced medication adherence, and increased engagement in cognitively and socially stimulating activities [13, 27, 42]. By addressing both the social context and the biological consequences of chronic stress, such interventions offer a more comprehensive strategy for promoting cognitive resilience and reducing long term risk for neurocognitive decline in this population [46, 48].

Likewise, policy efforts aimed at expanding access to mental and cognitive healthcare, funding HIV-specific support services, and confronting structural stigma in healthcare systems remain essential to long-term change [25, 29]. These efforts should include training for healthcare providers on implicit bias, intersectional stigma, and culturally responsive care, as well as broader advocacy for Medicaid expansion, integrated care models, and housing stability [51]. Importantly, solutions must be co-designed and co-led with those most affected—ensuring that BWLWH are not only the subjects of research but active participants in shaping their care and community resources [50].

By documenting the compounding effects of racism-gender-, and HIV-related discrimination on brain health, this study contributes to a deeper understanding of how social adversity may have biological implications. In doing so, it calls for urgent investment in multilevel strategies that recognize the full scope of stress experienced by BWLWH and center their lived experiences in the development of equitable health solutions. Ultimately, brain health equity for BWLWH will require both the dismantling of structural oppression and the amplification of community-based strengths, resilience, and healing practices.

Future research should examine whether cumulative exposure to intersectional psychosocial stressors contributes to aging-related cognitive decline and neurodegenerative processes, including Alzheimer’s disease [52]. Longitudinal and mechanistic studies will also be important for determining whether these associations differ across women and men and for clarifying the pathways through which social adversity may shape cognitive aging over time.

### Strengths and Limitations

This study offers several important strengths that contribute to the understanding of how intersectional adversity influences brain health outcomes among BWLWH. One of the key strengths is the adoption of an intersectional framework, which allowed for a comprehensive examination of how overlapping forms of discrimination—related to race, gender, and HIV status—collectively impact cognitive, behavioral, and functional health outcomes. By simultaneously examining multiple sources of psychosocial stress alongside structural determinants such as housing stability, access to healthcare, and economic insecurity, the study provides a multifaceted perspective on health disparities.

Another strength lies in the use of diverse and validated outcome measures. Health domains included cognitive function (via MoCA and Healthy Brain-9), sleep quality and behavior, dietary health, physical activity, and psychosocial functioning. This multidimensional approach allowed for a nuanced analysis of the pathways through which adversity manifests in health-related outcomes. Furthermore, the study’s integration of both frequency and appraisal-based measures of discrimination and microaggressions provides a deeper understanding of not only the occurrence of stigma but also the psychological toll it exerts on individuals. The use of both objective (e.g., cognitive assessments) and self-reported measures adds methodological depth, enabling a more comprehensive interpretation of participants’ lived experiences.

Despite these strengths, several limitations must be considered. First, the study relies on cross-sectional data, which precludes temporal and causal inference. Longitudinal research is needed to examine how these relationships evolve over time and to identify mediating or buffering factors such as social support, coping strategies, or neuroplasticity. Additionally, while the sample size is sufficient to detect significant associations, findings may not fully generalize beyond Black women living with HIV in similar sociocultural, geographic, and healthcare contexts; therefore, future work with larger and more geographically diverse samples will be important to enhance generalizability and capture the full spectrum of intersectional experiences among BWLWH, as well as to determine the broader applicability of these associations. The use of self-reported measures, particularly in assessing sensitive domains such as discrimination, sleep, and mental health, introduces the possibility of recall bias and social desirability effects. Although validated scales were used, participants may underreport or overreport certain behaviors or experiences based on social expectations. In addition, brain health was assessed using a combination of self-report and screening-based instruments and did not include neuroimaging, biomarker-based, or comprehensive neuropsychological methods, which may limit the precision with which neurological functioning was captured.

Moreover, the study may be subject to unmeasured confounding variables—such as trauma history, neurodevelopmental differences, or environmental exposures—that could influence cognitive and behavioral outcomes independent of intersectional adversity. Finally, while the quantitative data provide valuable insights into the associations between discrimination and brain health, the absence of qualitative data limits our ability to capture the nuanced, lived experiences of participants. Future research would benefit from a mixed-methods approach to deepen contextual understanding and better inform intervention design. While prior research has examined discrimination, stress, and neurological outcomes separately [1], the present study contributes to the literature by examining multiple, intersectional forms of psychosocial adversity in relation to brain health-related outcomes among Black women living with HIV.

Despite these limitations, the study makes a meaningful contribution to the literature on health disparities by centering the experiences of a population that is often underrepresented in cognitive health research. The findings underscore the importance of intersectional, community-based, and policy-driven solutions to promote brain health equity among Black women living with HIV. Clinically, these findings underscore the importance of integrating psychosocial screening into HIV care for Black women living with HIV, including routine assessment of discrimination-related stress, sleep difficulties, and mental health concerns. Neurocognitive complaints should be interpreted within the broader psychosocial context of patients’ lived experiences, and trauma-informed, culturally responsive models of care may be especially important for addressing the cumulative burden of intersectional stress. From a public health perspective, the findings support interventions that address both structural and interpersonal sources of harm, including anti-discrimination efforts within healthcare systems, expansion of supportive services, and community-based programs that strengthen resilience, belonging, and access to culturally affirming care. Multilevel strategies that address social determinants of health alongside psychological and cognitive well-being may be necessary to reduce inequities in brain health among Black women living with HIV. Continued research, grounded in both theory and lived experience, is essential to advancing health justice for this community.

## Conclusion

This study provides critical insight into the relationship between intersectional adversity and brain health among BWLWH, showing that experiences of discrimination—particularly racial, gender-based, and HIV-related—are significantly linked to poorer cognitive functioning, disrupted sleep, and reduced capacity for everyday activities. Using validated assessments and an intersectional framework, the study found that both the frequency and personal appraisal of discriminatory experiences meaningfully predicted health outcomes, indicating that the psychological toll of marginalization may be as impactful as its occurrence. Microaggressions across race, gender, and HIV status emerged as strong indicators of brain health related factors, with higher exposure consistently associated with lower cognitive and functional scores, greater reliance on sleep treatments, and more irregular sleep patterns, reinforcing and extending existing research on minority stress and allostatic load [46] by demonstrating how compounded social adversity becomes biologically embedded and drives disparities in neurological and behavioral health.

## Figures and Tables

**Table 1:** Sociodemographics among 183 BWLWH

Sociodemographic	*N*	%/SD
Income		
Less than $5,000	58	31.69%
$5,000 through $11,999	47	25.68%
$12,000 through $15,999	19	10.38%
$16,000 through $24,999	10	5.46%
$25,000 through $34,999	7	3.83%
$35,000 through $49,999	3	1.64%
$50,000 and greater	2	1.09%
Don’t Know	35	19.13%
Prefer Not to Answer	2	1.09%
Employment		
Full-Time Work	8	4.37%
Part-Time Work	18	9.84%
In School (Full/Part-Time)	6	3.28%
Neither Work nor in School	25	13.66%
On Disability	116	63.39%
Other Employment	4	2.19%
Education		
8th grade or lower	14	7.65%
Some High School	59	32.24%
High School/GED	58	31.69%
Some College	31	16.94%
College Graduate	11	6.01%
Some Graduate School	1	0.55%
Graduate School Degree	5	2.73%
Age Range	26–85	Years
Median Age	59	Years
Average Age	56.68	Years

*BWLWH* Black women living with HIV

**Table 2:** Regression outcomes: gendered racial microaggressions frequency and neurocognitive/sleep health controlling for medication adherence and mental health

Node	Node	F	*p*	Estimate	DF
Grms.freq.mean	hb9.score	3.95	0.015	1.53	(3, 161)
	night.sleep.score	4.53	0.024	0.89	(3, 161)
	sleep.treat.score	6.05	0.024	1.75	(3, 161)

All relationships reported in this table were statistically significant (*p* <.05). Analyses controlled for mental health diagnosis and self-reported medication adherence. *grms.freq.mean* = frequency of gendered racial microaggressions; *hb9.score* = Healthy Brain 9, a measure of everyday cognitive functioning; *night.sleep.score* = average nighttime sleep patterns; *sleep.treat.score* = frequency of sleep treatment or aid usage

**Table 3:** Regression outcomes: gendered racial microaggressions appraisal and neurocognitive/sleep health controlling for medication adherence and mental health

Node	Node	F	*p*	Estimate	DF
Grms.appraisal.mean	faqscore	1.93	0.101	0.78	(3, 107)
	hb9.score	3.82	0.006	1.67	(3, 107)
	night.sleep. score	5.14	0.043	0.69	(3, 107)
	sleep.treat. score	9.54	0.0003	2.39	(3, 107)

All relationships reported in this table were statistically significant (*p* <.05). Analyses controlled for mental health diagnosis and self-reported medication adherence. *grms.appraisal.mean* = cognitive appraisal of gendered racial microaggressions; *faqscore* = Functional Activities Questionnaire, which assesses daily living functioning; *hb9.score* = Healthy Brain 9, a measure of everyday cognitive functioning; *night.sleep.score* = average nighttime sleep patterns; *sleep.treat.score* = frequency of sleep treatment or aid usage

**Table 4:** Regression outcomes: total HIV microaggressions and neurocognitive/sleep health controlling for medication adherence and mental health

Node	Node	F	*p*	Estimate	DF
hivmicroaggression.total. mean	sleep. behav. score	4.34	0.067	0.28	(3, 162)
	hb9. score	6.93	0.0002	1.96	(3, 162)
	night. sleep. score	7.89	0.0002	1.22	(3, 162)
	sleep. treat. score	7.43	0.004	1.88	(3, 162)

All relationships reported in this table were statistically significant (*p* <.05). Analyses controlled for mental health diagnosis and self-reported medication adherence. *hivmicroaggression.total.mean* = total HIV-related microaggressions; *sleep.behav.score* = irregular or disrupted sleep behavior; *hb9.score* = Healthy Brain 9, a measure of everyday cognitive functioning; *night.sleep.score* = average nighttime sleep patterns; *sleep.treat.score* = frequency of sleep treatment or aid usage

**Table 5:** Regression Outcomes: Direct HIV microaggressions and neurocognitive/sleep health controlling for medication adherence and mental health

Node	Node	F	*p*	Estimate	DF
hivmicroaggression.direct. mean	sleep. behav. score	3.84	0.163	0.21	(3, 162)
	night. sleep. score	7.29	0.0005	1.13	(3, 162)
	sleep. treat. score	7.09	0.006	1.74	(3, 162)

All relationships reported in this table were statistically significant (*p* <.05). Analyses controlled for mental health diagnosis and self-reported medication adherence. *hivmicroaggression.direct.mean* = direct HIV-related microaggressions; *sleep.behav.score* = irregular or disrupted sleep behavior; *night.sleep.score* = average nighttime sleep patterns; *sleep.treat.score* = frequency of sleep treatment or aid usage

**Table 6:** Regression Outcomes: Indirect HIV microaggressions and neurocognitive/sleep health controlling for medication adherence and mental health

Node	Node	F	*p*	Estimate	DF
hivmicroaggression.indirect.mean	sleep. behav. score	4.20	0.086	0.24	(3, 162)
	hb9. score	4.19	0.012	1.23	(3, 162)
	night. sleep. score	6.4	0.002	0.95	(3, 162)
	sleep. treat. score	6.29	0.021	1.38	(3, 162)

All relationships reported in this table were statistically significant (*p* <.05). Analyses controlled for mental health diagnosis and self-reported medication adherence. *hivmicroaggression.indirect.mean* = indirect HIV-related microaggressions; *sleep.behav.score* = irregular or disrupted sleep behavior; *hb9.score* = Healthy Brain 9, a measure of everyday cognitive functioning; *night.sleep.score* = average nighttime sleep patterns; *sleep.treat.score* = frequency of sleep treatment or aid usage

**Table 7:** Regression Outcomes: Multi-discrimination scale of race and global health/neurocognitive/sleep health controlling for medication adherence and mental health

Node	Node	F	*p*	Estimate	DF
mds.race.total	globalhealth.avg	9.16	0.005	−0.10	(3, 161)
	faqscore	4.32	0.018	0.71	(3, 161)
	night.sleep.score	4.09	0.049	0.45	(3, 161)
	sleep.behav.score	6.12	0.004	0.30	(3, 161)
	sleep.treat.score	5.63	0.047	0.88	(3, 161)

All relationships reported in this table were statistically significant (*p* <.05). Analyses controlled for mental health diagnosis and self-reported medication adherence. *mds.race.total* = Multi-Discrimination Scale score for racial discrimination; *globalhealth.avg* = global health score representing overall physical and mental health; *faqscore* = Functional Activities Questionnaire, which assesses daily living functioning; *night.sleep.score* = average nighttime sleep patterns; *sleep.behav.score* = irregular or disrupted sleep behavior; *sleep.treat.score* = frequency of sleep treatment or aid usage. A negative estimate denotes a negative association

**Table 8:** Regression outcomes: multi-discrimination scale of gender and global health/neurocognitive/sleep health controlling for medication adherence and mental health

Node	Node	F	*p*	Estimate	DF	
mds.gender.total	globalhealth.avg	8.35	0.019	−0.10	(3, 162)	
	faqscore	5.22	0.005	1.03	(3, 162)	
	night.sleep.score	4.82	0.018	0.65	(3, 162)	
		sleep.behav.score	5.12	0.019	0.30	(3, 162)
	sleep.treat.score	5.92	0.038	1.12	(3, 162)	

All relationships reported in this table were statistically significant (*p* <.05). Analyses controlled for mental health diagnosis and self-reported medication adherence. *mds.gender.total* = Multi-Discrimination Scale score for gender-based discrimination; *globalhealth.avg* = global health score representing overall physical and mental health; *faqscore* = Functional Activities Questionnaire, which assesses daily living functioning; *night.sleep.score* = average nighttime sleep patterns; *sleep.behav.score* = irregular or disrupted sleep behavior; *sleep.treat.score* = frequency of sleep treatment or aid usage. A negative estimate denotes a negative association

**Table 9:** Regression outcomes: multi-discrimination Scale of HIV and global health/neurocognitive controlling for medication adherence and mental health

Node	Node	F	*p*	Estimate	DF
mds.hiv.total	globalhealth.avg	6.87	0.150	−0.04	(3, 161)
	faqscore	5.56	0.003	0.75	(3, 161)
	moca.score	3.03	0.010	−0.65	(3, 161)

All relationships reported in this table were statistically significant (*p* <.05), except for global health. Analyses controlled for mental health diagnosis and self-reported medication adherence. *mds.hiv.total* = Multi-Discrimination Scale score for HIV-related discrimination; *globalhealth.avg* = global health score representing overall physical and mental health; *faqscore* = Functional Activities Questionnaire, which assesses daily living functioning; *moca.score* = Montreal Cognitive Assessment, a screening tool for general cognitive performance. A negative estimate denotes a negative association

## Data Availability

Data is not available due to ongoing project aims.
